# Using practical health psychology approaches in your rheumatology practice

**DOI:** 10.1093/rap/rkaa026

**Published:** 2020-08-06

**Authors:** Keegan Knittle, Dominika Kwasnicka, Sebastian Potthoff, Ainslea Cross, Jenny Olson, Gill ten Hoor

**Affiliations:** r1 Faculty of Social Sciences, University of Helsinki, Helsinki, Finland; r2 Institute of Psychology, SWPS University of Social Sciences and Humanities, Wroclaw, Poland; r3 NHMRC CRE in Digital Technology to Transform Chronic Disease Outcomes, School of Population and Global Health, The University of Melbourne, Melbourne, Victoria, Australia; r4 Department of Social Work, Education and Community Wellbeing, Northumbria University, Newcastle upon Tyne; r5 Human Sciences Research Centre, University of Derby, Derby, UK; r6 Research and Evaluation, Diabetes Australia, Perth, Western Australia, Australia; r7 Faculty of Psychology and Neuroscience, Maastricht University, Maastricht, The Netherlands

People with rheumatic conditions face many challenges. These include coping with the emotional impact of debilitating chronic disease, overcoming physical symptoms, such as pain and fatigue, to carry out activities of daily living, and undertaking the self-management behaviours necessary to prevent disease progression and to limit symptoms (e.g., medication adherence, maintaining a physically active lifestyle) [1]. Each of these areas concerns the field of health psychology, which has generated a wealth of research findings on the patient-centred processes and clinical actions that can help patients to achieve positive outcomes.

Although health psychology-related issues are clearly important in managing rheumatic conditions, it is not always possible for clinicians to refer their patients to see a health psychologist. This often leaves health-care professionals to address these issues themselves. Unfortunately, many barriers limit the extent to which clinicians implement evidence-based health psychology-related interventions in their standard practice. This is often attributable to a lack of time, uncertainty regarding how and when to intervene, a lack of training or a lack of confidence in helping their patients through such issues [2, 3].

To support clinicians and allied health professionals in the use of evidence-based health psychology approaches and respond to the needs of their patients and clients, even when time and resources are limited, the European Health Psychology Society has launched the Practical Health Psychology blog (PracticalHealthPsychology.com). In it, leading researchers in the field of health psychology write very brief blog posts that summarize their research findings and provide readers with clear recommendations for practice. Each blog post is then translated into 27 different languages and disseminated worldwide by a team of dedicated National Editors. The blog is currently published in English, Bulgarian, Mandarin Chinese, Croatian, Czech, Danish, Dutch, Finnish, French, German, Greek, Hebrew, Indonesian, Italian, Korean, Latvian, Lithuanian, Malaysian, Polish, Portuguese, Romanian, Russian, Slovenian, Spanish, Swedish, Turkish and Ukrainian.

To illustrate the relevance of the practical health psychology blog, let us assume that one of your patients seems to be non-adherent to DMARD therapy. Readers of Professor Kerry Chamberlain's [4] blog post on medication adherence would recognize that the patient's social environment could be the key limiting factor to adherence and that understanding this social context is a crucial first step to improving adherence. Additional posts have covered topics including e-health interventions, discussing weight loss, planning interventions, the importance of self-efficacy, physical activity motivation, financial incentives for change in health behaviour, habits, goal setting, mental imagery and more. Each of these posts contains practical tips on how to leverage health psychology to help your rheumatology patients.

Concise blog posts, with case examples and a clear focus on the techniques of practical implementation, offer clinicians a valuable resource to improve their clinical care and assist their patients optimally in self-managing rheumatic diseases. PracticalHealthPsychology.com provides these to clinicians and allied health professionals from all backgrounds free of charge, and we hope they will help you to implement insights from health psychology into your rheumatology practice.

*Funding:* No specific funding was received from any funding bodies in the public, commercial or not-for-profit sectors to carry out the work described in this manuscript.

*Disclosure statement:* The authors are all members of the editorial board of the Practical Health Psychology blog, which is a non-profit initiative of the European Health Psychology Society. The authors declare no further potential conflicts of interest.
